# A Preregistered, Prospective, Longitudinal Examination of the Impact of Self-Initiated Sexual Orientation Change Efforts on Health and Changes in Sexuality

**DOI:** 10.1007/s10508-026-03517-y

**Published:** 2026-08-15

**Authors:** G. Tyler Lefevor, Christopher H. Rosik, A. Lee Beckstead, Ty R. Mansfield, Mark A. Yarhouse

**Affiliations:** 1 Department of Psychology, Utah State University, 6405 Old Main Hill, Logan, UT 84322 USA; 2 Link Care Center, Fresno Pacific University, Fresno, CA USA; 3 Private Practice, Salt Lake City, UT USA; 4 Department of Church History and Doctrine, Brigham Young University, Provo, UT USA; 5 Department of Psychology, Wheaton College, Wheaton, IL USA

**Keywords:** Sexual orientation change efforts, Depression, Sexual fluidity, LGBTQ +, Sexual orientation

## Abstract

Although sexual orientation change efforts are generally understood to be ineffective and health-undermining, this understanding is based almost entirely on retrospective, cross-sectional data. We present prospective, longitudinal data from a sample of 315 sexual minority current/former members of the Church of Jesus Christ of Latter-day Saints to examine the efficacy and impact of self-initiated sexual orientation change efforts (SISOCE). We found that 74.6% of participants engaged in SISOCE at some point in their lives. Of the 13.7% of participants who were engaged with SISOCE at the start of the study, 62.8% terminated their SISOCE in the 2-year window of the study. We failed to find significant differences in sexual attraction, aversion, or behavior two years later between those who were actively engaged in SISOCE and those who were not. At baseline, participants engaged in SISOCE reported more religiousness, self-alienation, desires to be exclusively heterosexual (i.e., internalized homonegativity), and concealment than participants not engaged in SISOCE. We also found that participants engaged in SISOCE reported less social support, well-being, and self-rated health alongside more depression than participants not engaged in SISOCE at baseline. Two years later, participants who engaged in SISOCE at baseline reported more depression and less social support than participants not engaged in SISOCE. Results support the idea that trying to change one’s sexual orientation is health-undermining and ineffective with caveats on how health-undermining, whether we know it is ineffective for everyone, and whether it is always health-undermining. We recommend therapists provide acceptance-focused and person-affirming assessments/interventions that promote identity development.

**Supplementary Information:**

The online version contains supplementary material available at https://doi.org/10.1007/s10508-026-03517-y.

## Introduction

After two years of literature review and discussion, the American Psychological Association’s (APA) Task Force on Appropriate Therapeutic Responses to Sexual Orientation (2009) concluded that “none of the recent research [on sexual orientation change efforts] from 1999 to 2007 meets methodological standards that permit conclusions regarding efficacy or safety” (p. 2). They further concluded that existing literature suggested that “individuals experienced harm from sexual orientation change efforts” and that “it is unlikely that individuals will be able to reduce same-gender attractions or increase other-sex sexual attractions through sexual orientation change efforts” (p. 2). This report was critical in informing the APA’s (2021) resolution denouncing sexual orientation change efforts, a position echoed by nearly all other major mental health professional organizations. Findings from reports like those issued by the APA Task Force’s (2009, 2021) along with advocacy efforts have prompted 27 states to enact bans on therapeutic practices to change sexual orientation among minors (Movement Advancement Project, 2024).

This near consensus among professional organizations and the bans enacted by multiple state legislatures have led many to believe that widespread and conclusive empirical support shows sexual orientation change efforts are ineffective and harmful. However, as indicated by the APA Task Force’s (2009) summary, there are actually few methodologically sound, quantitative studies about the health impacts or efficacy of sexual orientation change efforts. Specifically, nearly all studies about sexual orientation change efforts (1) are based on restricted, self-selected samples with heterocentric biases, (2) do not attempt to account for impression management, (3) do not manage researcher biases, (4) do not use comparison or control groups, (5) are cross-sectional, and/or (6) are based on therapist impressions of client outcomes (Beckstead, 2012). These limitations substantially restrict the conclusions that may be drawn, which can undermine both the public and lawmakers’ confidence in the scientific basis for the policies advocated by professional organizations.

The issue of change remains central to both political and scientific discussions. Thus, although the efficacy and health impacts associated with change efforts have been investigated in qualitative and correlational studies, methodologically rigorous studies on change efforts are as important as ever. To address this need and correct many of the missteps of earlier research, we assembled an ideologically diverse research team to conduct a preregistered study of the impact of current self-initiated sexual orientation change efforts on health and changes in sexuality. We thus present data from a 2-year, prospective, longitudinal sample of 315 sexual minority participants with a diversity of experiences around sexual orientation change efforts.

## What Is Sexual Orientation Change Efforts and How Is Self-Initiated Sexual Orientation Change Efforts Different?

Sexual orientation change efforts (SOCE) is an umbrella term characterized by individuals’ actions to reduce their same-gender sexual attractions and behaviors and/or foster other-gender sexual attractions and behaviors (see Lefevor et al., 2022a, 2022b for a discussion of sexual concordance and discordance and importance of distinguishing between sexual attraction, behavior, and identity). SOCE describe a wide variety of behaviors including religious and spiritual practices (e.g., personal righteousness, prayer, church counseling, exorcisms), personal practices (e.g., avoiding people who experience same-gender attraction or those to whom one is attracted, denying experiencing same-gender attraction, employing strict rules for oneself around sexual behavior and exploration, suppressing same-gender sexual thoughts, pairing same-gender desires with negative self-talk or self-punishment), psychotherapeutically backed practices (e.g., attending group therapy for people pursuing change, processing traumatic events, using aversive conditioning), and practices unique to SOCE (e.g., enforcing stereotypical gender roles; encouraging heterosexual relationships while discouraging same-gender relationships; framing same-gender attraction as a phase, an addiction, admiration, and/or the result of poor parenting and trauma; Dehlin et al., 2015; Glassgold, 2022; Salway et al., 2025).

Although discussion about SOCE is often thought to refer to therapist-assisted change efforts, the majority of SOCE are not therapist-assisted. Individual effort, personal righteousness, and church counseling are all more commonly employed SOCE than individual or group psychotherapy (Dehlin et al., 2015). Further, although early literature on SOCE examined therapist-led SOCE, often through aversive conditioning or shock therapy (Beckstead, 2012), all studies of SOCE in the last two decades have relied on individual self-report data (i.e., no clinical trials) and have most often examined SOCE broadly (Glassgold, 2022).

Given the shift away from clinical trial research and because the language of SOCE so clearly conjures mental images of psychotherapy, we suggest that the language of self-initiated sexual orientation change efforts (SISOCE) more accurately describes the recent scientific literature on SOCE. The language of SISOCE more clearly emphasizes individuals’ decision to seek change rather than others’ efforts to change an individual. Thus, we conceptualize SISOCE as individuals’ efforts to change, which are influenced by an individual’s context, including social policies, religious milieus, and interpersonal relationships.

### Key Findings from Studies of SOCE and SISOCE

When taken together, studies on SOCE and SISOCE converge in a handful of meaningful ways. Firstly, they suggest that many if not most individuals who seek change find SISOCE health-undermining (though there are notable exceptions; e.g., Jones & Yarhouse, 2011). Secondly, they suggest that purposefully pursued experiences of sexual orientation change (as opposed to naturalistic change and fluidity) are unlikely to result in success.

#### SISOCE Is Health-Undermining for Many and Likely Most Participants

It is clear that many if not most sexual and gender minority people who have been studied report psychological distress from their engagement with SISOCE. Several cross-sectional studies have examined SISOCE internationally, finding SISOCE to be linked with depressive symptoms, self-injury, and suicidality in South Korea (Lee et al., 2021), Colombia (del Río-González et al., 2021), Canada (Salway et al., 2020), New Zealand (Fenaughty et al., 2023), Malaysia (Tan et al., 2025), and the USA (Blosnich et al., 2020; Dehlin et al., 2015; Green et al., 2020; Tran et al., 2024). Qualitative studies detail the emotional distress experienced by many engaged with SISOCE, including religious conflict, self-loathing, depression, weight gain, and suicidality (Beckstead & Morrow, 2004; Shidlo & Schroeder, 2002). A small number of studies have found no harm on average to participants and, in some cases, benefits from SISOCE. These participants were typically conservatively religious (Byrd et al., 2008; Jones & Yarhouse, 2011; Rosik et al., 2021). Some studies reported that benefits were secondary to the goal of change, such as experiencing greater self-acceptance or increased social support (APA, 2009; Chan et al., 2022).

#### Change in Sexual Orientation Due to Engagement with SISOCE Is Unlikely

For most people who engage with SISOCE, SISOCE appear to be ineffective in changing their orientation (Comer et al., 2024; Przeworski et al., 2021). Few people engaged with SISOCE report sexual orientation change, and many studies fail to find evidence that SISOCE produce shifts in sexual orientation (Maccio, 2011; Schaeffer et al., 2000). At the same time, at least some people report changes in their sexual orientation that they attribute to SISOCE (Byrd et al., 2008; Nicolosi et al., 2000). These changes appear to happen when individuals first engage with SISOCE (Jones & Yarhouse, 2011), are more common among cisgender men who are married to cisgender women (Sullins & Rosik, 2025), and may in some cases be understood as shifts within a fluid or bisexual orientation, changes in individuals’ views of their identities, or abstinence from same-gender sexual contact (Byrd et al., 2008; Schaeffer et al., 1999; Vasey et al., 2003).

### Key Vulnerabilities of Studies of SISOCE

The APA Task Force (2009) and all reviews of SISOCE research (Beckstead, 2012; Przeworski et al., 2021; Rosik, 2023) have emphasized that nearly all research on SISOCE is not methodologically rigorous. We briefly unpack how these limitations impact studies’ findings.

#### Most Studies Are Retrospective

Most studies ask participants if they have ever experienced SOCE and then examine differences between those who have and have not experienced SOCE (Blosnich et al., 2020; Fenaughty et al., 2023; Salway et al., 2020). SISOCE studies based on retrospective reports are problematic because one’s recollection of the past can be altered by one’s present experience. For example, participants who stopped pursuing change may now view those efforts as particularly health-undermining (e.g., Shidlo & Schroeder, 2002) and participants continuing to engage with change may see those efforts as quite helpful (Byrd et al., 2008; Rosik, et al., 2023; Sullins & Rosik, 2025). Prospective SISOCE studies (e.g., Jones & Yarhouse, 2011) have documented that at least some individuals report change, though this group still appears to be a minority.

#### Nearly All Studies Are Cross-Sectional

With the notable exception of two limited longitudinal studies (Jones & Yarhouse, 2011; Pela & Sutton, 2021), all studies of SISOCE are cross-sectional, meaning that causal attributions are difficult to make. When an individual engages with SISOCE because doing so aligns with their heteronormative religious beliefs or heterosexual relationship, engaging with SISOCE can bring perceptions of both improved health and changes in sexual orientation since SISOCE can encourage connection with these beliefs and relationships. Conversely, when an individual rejects SISOCE because doing so aligns with an individual’s homonormative religious beliefs or same-gender relationship, individuals may overattribute past pain to SISOCE.

#### Many Studies Recruit Self-Selecting Samples

Although more representative studies on SISOCE have been done in the last decade (Blosnich et al., 2020; Fenaughty et al., 2023; Salway et al., 2020), many SISOCE studies recruit participants from organizations with a particular viewpoint, limiting ideological generalizability. When researchers primarily recruit from lesbian, gay, bisexual, transgender, and queer/questioning (LGBTQ+) organizations, participants are likely to be liberal, younger, hold more negative attitudes toward religion, and be at earlier stages in their sexual identity development (Lefevor et al., 2021). Conversely, when researchers primarily recruit from religious organizations, participants they recruit are likely to be conservative, older, White, and cisgender men (e.g., Byrd et al., 2008; Jones & Yarhouse, 2011; Sullins & Rosik, 2025).

#### Few Studies Employ Comparison Groups

Many SISOCE studies examine a homogenous group of participants who are all trying to change, without including a reference group of participants not trying to change (e.g., Jones & Yarhouse, 2011; Shidlo & Shroeder, 2002). Without a comparison group, it is unclear whether changes or health impacts are related to engagement with SISOCE or other factors.

#### Perhaps All Studies Have Been Conducted by Ideologically Homogenous Research Teams 

We are unaware of any research on SISOCE that has explicitly been conducted with an ideologically diverse research team. Such a team would include socio-politically conservative, moderate, and liberal members with differing experiences and perspectives about what constitutes SOCE, whether SOCE can ever be helpful or less harmful for same-gender attracted individuals, and, if so, for whom. Researchers’ assumptions about what is worth investigating and how best to investigate it influence their studies and the results they find. Furthermore, researchers may be hesitant to report findings that could potentially be upsetting to their scholarly peer group or the organizations that have provided them with support, data, and/or funding. Consequently, teams can set up studies that make it easier to show how SISOCE undermines health or how SISOCE may effectuate change. All of these problems are further compounded by the lack of preregistered hypotheses, allowing researchers flexibility in determining which analyses they will present.

### The Present Study

To address the limitations of the vast majority of SISOCE research, we assembled an ideologically diverse research team to conduct a preregistered study of the impact of current SISOCE on health and changes in sexuality. Our ideologically diverse research team is comprised of researchers who are both LGBTQ+ and cisgender and heterosexual, politically conservative and liberal, and religious and nonreligious. We work in a variety of settings (faculty at religious institutions, faculty at nonreligious institutions, private practice, faith-based therapy settings) and have different experiences and lenses on SISOCE. Parts of our team have worked together on other “adversarial” collaborations (Lefevor et al., 2019; Rosik et al., 2021, 2022) while others are newer and were recruited to bring enhanced ideological diversity to the team.

We use data from a 2-year, prospective, longitudinal sample of 315 sexual minority current and former members of the Church of Jesus Christ of Latter-day Saints (CJCLDS) with diverse experiences around SISOCE. Current/former members of the CJCLDS are more likely than other sexual minority individuals generally to have had exposure to SISOCE (Dehlin et al., 2015); thus, we suspected that we would more readily be able to find participants with substantial SISOCE exposure without specifically recruiting participants because of SISOCE engagement. The 2-year window was selected to take a broader life trajectory view of the impacts of SISOCE engagement.

Based on our understanding of the literature, we examine five core research questions. Later on in the paper, we describe the analytic approaches used to assess each research question in Table 1.

- Research Question 1: What percentage of participants who were trying to reduce, change, or eliminate their same-gender sexuality is still trying to do so two years later?
- Research Question 2: How do participants who are still trying to reduce, change, or eliminate their same-gender sexuality differ from those who are no longer trying in demographic variables, religiousness, support, and sexuality-related variables?
- Research Question 3: How do participants who are still trying to reduce, change, or eliminate their same-gender sexuality differ from those who are no longer trying in their health?
- Research Question 4: How do participants who are still trying to reduce, change, or eliminate their same-gender sexuality differ from those who are no longer trying in their religiousness, support, sexuality, and health two years later?
- Research Question 5: Do participants who are still trying to reduce, change, or eliminate their same-gender sexuality differ from those who are no longer trying in the degree of change they report in their same-gender and other-gender sexual attraction, sexual aversion, and sexual behavior two years later?

**Table 1:** Research questions and the samples and analytic approaches used to answer them

Research question	Sample used	Analytic approach
1. What percentage of participants who were trying to reduce, change, or eliminate their same-gender sexuality is still trying to do so two years later?	Both Wave I and Wave II	Examine what the percentage of participants who report Wave I SISOCE also report Wave II SISOCE
2. How do participants who are still trying to reduce, change, or eliminate their same-gender sexuality differ from those who are no longer trying in demographic variables, religiousness, support, and sexuality-related variables?	Only Wave I	(1) Separate participants into two groups (reported SISOCE at Wave I, did not report SISOCE at Wave I) (2) MANOVA for religiousness variables measured at Wave I (3) MANOVA for support variables measured at Wave I (4) MANOVA for sexuality-related variables measured at Wave I
3. How do participants who are still trying to reduce, change, or eliminate their same-gender sexuality differ from those who are no longer trying in their health?	Only Wave I	(1) Separate participants into two groups (reported SISOCE at Wave I, did not report SISOCE at Wave I) (2) MANOVA for health-related variables measured at Wave I
4. How do participants who are still trying to reduce, change, or eliminate their same-gender sexuality differ from those who are no longer trying in their religiousness, support, sexuality, and health two years later?	Both Wave I and Wave II	(1) Separate participants into two groups (reported SISOCE at Wave I, did not report SISOCE at Wave I) (2) MANOVA for religiousness variables measured at Wave II (3) MANOVA for support variables measured at Wave II (4) MANOVA for sexuality-related variables measured at Wave II
5. Do participants who are still trying to reduce, change, or eliminate their same-gender sexuality differ from those who are no longer trying in the degree of change they report in their same-gender and other-gender sexual attraction, sexual aversion, and sexual behavior two years later?		(1) Separate participants into two groups (reported SISOCE at Wave I, did not report SISOCE at Wave I) (2) MANOVA for health-related variables measured at Wave II

## Method

### Participants and Procedure

To be included in the overarching longitudinal study, participants must have (1) reported experiencing some degree of same-gender attraction, engaging in same-gender sexual behavior, endorsing an LGBTQ+ or same-gender attracted identity, and/or experiencing discordance between their sex assigned at birth and the gender identity typically associated with that sex; (2) reported being baptized a member of the Church of Jesus Christ of Latter-day Saints at some point in their lives; (3) been 18 years or older; (4) resided in the USA; and (5) been willing to have the research team reach out to them for follow-up surveys. A total of 567 participants met the inclusion criteria in 2022 and form the core sampling pool of this present study.

To be included in the present study, participants must have (1) been part of the overarching longitudinal study, (2) responded to questions inquiring about sexual orientation change efforts (27 did not respond to these questions), and (3) provided data in 2024 about at least one focal variable. A total of 540 participants from 2022 were eligible in 2024.

In 2020, we employed a comprehensive community sampling approach. Participants were thus recruited through (1) posts by the research team in key social media groups for SGM Latter-day Saints (e.g., North Star, Affirmation), (2) posts made by prominent LDS SGM community leaders on their personal social media accounts, (3) descriptions of the survey in news outlets (e.g., Salt Lake Tribune, the Cache Valley Herald), (4) appearances by the research team on podcasts listened to by SGM Latter-day Saints (e.g., Listen, Learn, Love; Mormon Stories), (5) advertisements by therapeutic organizations that serve SGM Latter-day Saints (Flourish Therapy, college counseling centers in Utah), and (6) snowball sampling.

Because the 2020 wave included so few participants who were still affiliated with the Church of Jesus Christ of Latter-day Saints and because this wave occurred during the COVID-19 pandemic, additional participants were recruited in 2022. These participants were recruited using the same comprehensive community sampling approach used in 2020.

In 2022 and 2024, participants were recruited from the extant list of existing participants who participated in 2020 or 2022. All participants on this list have indicated their consent to receive follow-up survey invitations. In January 2024, the research team contacted these participants to invite them to participate again in the survey. Participants were compensated $10 in 2020 and 2022 and $25 for their participation in 2024. Each participant received up to three emails containing a Qualtrics link inviting them to participate in 2024. Given the limitations of 2020 data, the present study uses data solely from 2022 and 2024.

### Measures

Participants completed basic demographic information about age, sex, gender, race/ethnicity, sexual identity, and education. All constructs except desirable responding (only in 2024) were assessed in 2022 and 2024. The only measures created for this study (or rather the larger longitudinal study) were measures of (1) SISOCE, (2) sexual attraction, and (3) sexual aversion. Note that all measures are displayed in Table 3, indicating which measures were administered at which time points, with dashes indicating that a measure was not administered at a particular time point.

#### Desirable Responding

Participants completed the Marlow–Crowne Social Desirability Short Form (Loo & Thorpe, 2000; Reynolds, 1982) to assess socially desirable responding. This measure presents 13 self-referential statements such as, “No matter who I’m talking to, I’m always a good listener,” that participants judge as *True* or *False*. All items represent desirable responding, and negatively worded items were reverse coded.

#### Measures of Religiousness

##### Affirming and Cis/Heteronormative Religious Beliefs

Affirming and cis/heteronormative Latter-day Saint beliefs were measured at both waves using a scale developed by McGraw et al. (2023). Using a scale ranging from *Strongly disagree* (1) to *Strongly agree* (5), participants indicated their agreement with five “affirmative” LDS beliefs (e.g., “It is part of my Heavenly Father’s plan for me to be LGBTQ”) and ten cis/heteronormative beliefs (e.g., “I must have done something wrong in the pre-earth life to be LGBTQ in this life”).

##### Church of Jesus Christ of Latter-day Saints (CJCLDS) Connectedness

Connectedness with CJCLDS communities was measured at both waves using a modified version of Frost and Meyer’s (2012) Community Connectedness scale. Participants indicated their agreement with seven statements (e.g., “You feel a bond with your local ward or branch”) from *Disagree strongly* (1) to *Agree strongly* (4).

##### Service Attendance

Service attendance was measured at both waves using the organizational religious behavior item from the Duke University Religiousness Index (Koenig & Büssing, 2010). This item asks participants to report how often they attend church or other religious meetings, with responses ranging from *Never* (1) to *More than once a week* (6).

#### Measures of Support

##### Family and Friend Support

Family and friend support were measured at both waves using the Family Support and Friend Support subscale of the Multidimensional Scale of Perceived Social Support (Zimet et al., 1988). Participants indicated their agreement with four items (e.g., “I get the emotional help and support I need from my family,” “I can count on my friends when things go wrong”) from *Strongly disagree* (1) to *Strongly agree* (5).

#### Sexuality-Related Variables

##### Self-Initiated Sexual Orientation Change Efforts (SISOCE)

Participants’ SISOCE were measured through the question, “How much time have you spent trying to reduce, change, and/or eliminate your same-sex sexual attractions, behaviors, or orientation (for example, using avoidance, denial, self-control, religion/faith, or counseling programs).” Participants reported the age at which they “started” and “ended” trying to change. Participants’ reports of their SISOCE were independent of whether they engaged in organized efforts with a group, program, or professional (which we did not assess).

##### Same-Gender and Other-Gender Sexual Attraction and Aversion

At both waves, participants were asked to indicate to what degree they currently experience sexual attraction and sexual aversion to men and women using the following scale: *None* (0%), *Mild* (around 17%), *Some* (around 33%), *Medium* (around 50%), *Moderate* (around 67%), *Strong* (around 83%), and *Very Strong* (around 100%). No further instruction was provided to participants about what we meant about sexual attraction or sexual aversion, thus relying on participants’ intuitive understanding of these constructs. Responses were then coded based on the participant’s gender to create variables for same-gender attraction, same-gender aversion, other-gender attraction, and other-gender aversion. Participants who identify as transgender, gender nonbinary, and gender expansive (TNB) were coded as missing for these variables.

##### Same-Gender and Other-Gender Sexual Behavior

At both waves, same-gender sexual behavior and other-gender sexual behavior were measured using the Center for Disease Control and Prevention’s (2013) sexual behavior questions from the Youth Risk Behavior Survey. Participants were asked to indicate how often in the past year they engaged in oral, anal, or vaginal sex with men and how often in the past year they engaged in oral, anal, or vaginal sex with women. Responses were then coded based on the participant’s gender to create variables for same-gender sexual behavior and other-gender sexual behavior. Participants who identify as TNB were coded as missing for these variables.

##### Internalized Homonegativity

Internalized homonegativity was measured at both waves using the Internalized Homonegativity subscale from the Lesbian, Gay, and Bisexual Identity Scale (Mohr & Kendra, 2011). This scale was chosen based on its classification in a recent meta-analysis of measures of internalized homonegativity as a widely used scale that conceptualizes internalized homonegativity orthogonally from distress (Lefevor et al., 2023a, 2023b). Participants were asked to indicate their agreement with three items (e.g., “I believe it is unfair that I am attracted to people of the same sex”) using a 6-point Likert scale ranging from *Disagree strongly* (1) to *Agree strongly* (6). Indeed, all items of this scale conceptualize internalized homonegativity as desires to be exclusively heterosexual.

##### Outness

Outness was measured at both waves using a single item: “How open/out are you about your experience with same-sex attraction (current or former) and/or being LGBT + ” (Wilkerson et al., 2016). Participants were asked to indicate their degree of openness to others on a scale ranging from *Not at all open (out)* (1) to *Open (out) to all or most people I know* (5).

##### Self-Alienation

Self-alienation was measured at both waves using the Self-Alienation subscale of the Authenticity Scale (Wood et al., 2008). Participants were asked to indicate how well a series of 4 statements (e.g., “I feel as if I don’t know myself very well”) describe them, using a scale ranging from *Does not describe me at all* (1) to *Describes me very well* (7).

#### Measures of Health

##### Depression

Depression was measured at both waves using the 9-item version of the Physicians Health Questionnaire (Kroenke et al., 2001). Participants were asked to indicate how often they have been bothered by various depressive symptoms over the past two weeks (e.g., “feeling tired and feeling down, depressed, or hopeless”) on a 4-point Likert scale ranging from *Not at all* (0) to *Nearly every day* (3).

##### Suicidality

Suicidality was measured at both waves using the four-item Suicide Behaviors Questionnaire-Revised (SBQ-R; Osman et al., 2001). Participants were asked to indicate lifetime suicide thoughts/attempts, suicidal thoughts in the past year, and estimated future possibility of suicidal behaviors.

##### Flourishing

Flourishing was measured at both waves using different scales. In 2022, it was measured using the 15-item, PERMA Profiler (Butler & Kern, 2016). Participants were asked to indicate how often they experience a number of statements (e.g., “In general, how often do you feel joyful?”) on a 10-point Likert scale ranging from *Never* (1) to *Always* (10). In 2024, it was measured using the Flourishing Scale (Diener et al., 2010). Participants were asked to indicate their agreement with eight items related to well-being (e.g., “I lead a purposeful and meaningful life”) using a scale ranging from *Strongly disagree* (1) to *Strongly agree* (7).

##### Self-Rated Health

Self-rated health was measured using the question, “Would you say that in general your health is …” on a 5-point scale ranging from *Poor* (1) to *Excellent* (5). This question is the most used question to assess self-reported health (Hays et al., 2015).

### Analysis Plan

Scaled variables were created by averaging participants’ responses across the items of the scale. All scaled variables evidenced acceptable skewness and kurtosis (less than -4 or greater than 4). Missing data on scaled variables were treated using casewise deletion only for the analyses for which data were missing.

The key “SISOCE” (self-initiated sexual orientation change efforts) variable was created by subtracting participants’ age from the age at which they stopped trying to reduce, change, and/or eliminate their same-gender sexuality. Through this process, we created a 2022 and 2024 SISOCE variable, considering participants with a value of “0” (indicating that they are still trying) or “1” (indicating that they stopped trying within the last year and may thus still be actively impacted from having tried) to be “still trying” and participants with any other positive value to be “not trying.” A single participant with a negative value for 2022 SISOCE (i.e., reported ending before beginning SISOCE) was removed from analyses. We describe all research questions and the approaches used to answer them in Table 1.

#### Research Question 1

We evaluated Research Question 1 by examining what percentage of participants who report Wave I SISOCE also report Wave II SISOCE.

#### Research Question 2

We evaluated Research Question 2 entirely using 2022 data by creating two groups: participants who reported ongoing or recently terminated SISOCE in 2022 and participants who did not report ongoing or recently terminated SISOCE in 2022. We evaluated demographic differences between these two groups in gender, race/ethnicity, and religious affiliation using Chi-squared analyses, and we evaluated differences in age and in desirable responding by using independent samples *t*-tests, controlling for any significant differences in subsequent analyses.

We then evaluated differences between the two groups in religiousness, support, and sexuality variables by using multivariate analyses of variance (MANOVA). We conducted three MANOVAs, each using the two SISOCE groups as the independent variable and one cluster of dependent variables: religiousness (Service Attendance, Affirming and Cis/heteronormative LDS Beliefs, CJCLDS Connectedness), support (Family Support, Friend Support), and sexuality (Same-Gender Attraction, Other-Gender Attraction, Outness, Internalized Homonegativity, Self-Alienation). Where a MANOVA was significant, we conducted Tukey’s post hoc tests to determine which dependent variables had significant differences.

#### Research Question 3

We evaluated Research Question 3 using 2022 data with our same two groups of ongoing or recently terminated SISOCE and no ongoing or recently terminated SISOCE. We conducted a single MANOVA with SISOCE group as the independent variable and four key outcomes as dependent variables: Depression, Suicidality, Subjective Well-Being, and Self-Rated Health. If the omnibus MANOVA was significant, we conducted Tukey’s post hoc tests to determine which dependent variables had significant differences.

#### Research Question 4

We evaluated Research Question 4 by replicating the analyses done with Research Questions 2 and 3 using 2024 variables as dependent variables rather than 2022 variables. As such, we conducted four MANOVAs with four clusters of dependent variables: religiousness (Service Attendance, Affirming and Cis/heteronormative LDS Beliefs, CJCLDS Connectedness), support (Family Support, Friend Support), sexuality (Same-Gender Attraction, Other-Gender Attraction, Outness, Internalized Homonegativity, Self-Alienation), and health (Depression, Suicidality, Subjective Well-Being, Self-Rated Health). Where a MANOVA was significant, we conducted Tukey’s post hoc tests to determine which dependent variables had significant differences.

#### Research Question 5

To evaluate Research Question 5, we first created variables representing changes in same- and other-gender attraction, aversion, and sexual behavior. We subtracted 2022 values from 2024 values to create six variables: Change in Other-Gender Sexual Attraction, Change in Other-Gender Sexual Aversion, Change in Other-Gender Sexual Behavior, Change in Same-Gender Sexual Attraction, Change in Same-Gender Sexual Aversion, and Change in Same-Gender Sexual Behavior. We then conducted a MANOVA using 2022 SISOCE groups as the independent variable and these six change variables as dependent variables. Where an omnibus MANOVA was significant, we conducted Tukey’s post hoc tests to determine which dependent variables had significant differences. We also report the percentage identifying as LGBTQ+ in 2022 and the percentage identifying as LGBTQ+ in 2024.

## Results

A total of 567 individuals participated in 2022, of which 322 participated again in 2024, yielding a 57% retention rate. Of those 322, 3 failed to provide data about their SISOCE in 2022 and 4 failed to provide data about their SISOCE in 2024. Thus, we have four slightly differentially sized analytic samples: (a) all participants (*n* = 322), (b) 2022 participants (*n* = 319), (c) 2024 participants (*n* = 318), and (d) participants with SISOCE data at both waves (*n* = 315). To maximize our use of available data, we used the largest sample possible to answer each research question (e.g., using group “b” to answer research questions about 2022 rather than group “d”). Our largest possible sample of participants was predominantly White (94.1%), gay/lesbian (59.0%), 20–50 years old (*M*_2022Age_ = 34.42, *SD* = 12.15), formally educated (74.9% with a bachelor’s degree or more) cisgender men (59.6%) and women (25.5%). Only 25.4% of participants reported never engaging in SISOCE. See Table 2.

**Table 2:** Participant demographic characteristics in 2022 unless otherwise noted

Gender			Sexual Identity in 2022	
	Cisman	59.6%	Gay/Lesbian	59.0%
	Ciswoman	25.5%	Bisexual/Pansexual	15.8%
	Transman	0.9%	Queer/Other	10.6%
	Transwoman	1.2%	Same-Gender Attracted	7.7%
	Nonbinary/genderqueer	12.7%	No Label	2.8%
Race/Ethnicity			Asexual	4.0%
	Asian/Asian American	0.3%	Sexual Identity in 2024	
	Latine/Hispanic American	1.2%	Gay/Lesbian	63.4%
	Native Hawaiian/Pacific Islander	0.3%	Bisexual/Pansexual	15.2%
	White/Caucasian	94.1%	Queer/Other	9.3%
	Multiethnic/Other	4.0%	Same-Gender Attracted	5.9%
Religious Affiliation			No Label	1.6%
	None/Unaffiliated	40.7%	Asexual	4.7%
	Catholic/Christian	2.8%	Education	
	Latter-day Saint	50.6%	High School or Less	2.2%
	Other	5.9%	Some College	23.0%
			Bachelor’s Degree	42.9%
			Graduate Degree	32.0%

We examined how participants who returned for the 2024 survey (*n* = 322) differed from those who took the 2022 survey but did not take the 2024 survey (*n* = 245). We did not detect differences in Age (*t* = 0.68, *p* = .50) or Sexual Identity (χ^2^(5) = 10.23, *p* = .07, Cramer’s V = .13). However, we found that we failed to retain TNB participants (21.4% of 2022 sample but only 15.5% of 2024 sample; χ^2^(5) = 27.36, *p* < .01, Cramer’s V = .22) and participants of color (13.7% of 2022 sample but only 5.6% of 2024 sample; χ^2^(5) = 28.79, *p* < .01, Cramer’s V = .23).

Of the 43 participants who reported ongoing or recently terminated SISOCE in 2022, 16 reported ongoing or recently terminated SISOCE in 2024 (37.2%). It appears that recent SISOCE was a relatively rare event among participants (13.7%; 43 of 315 participants) and that few participants sustained SISOCE, though these findings should be interpreted within the unique characteristics of our sample (e.g., a raised religious sample that primarily identified as LGBQ+).

### Assumption Checking

All variables evidenced acceptable skewness and kurtosis (values between −4 and 4). We report the means, standard deviations, and internal consistencies of all study variables in Table 3.

**Table 3:** Means, standard deviations, and internal consistencies of study variables

	Range	2022 Wave			2024 Wave		
		*M*	*SD*	*α*	*M*	*SD*	*α*
1. Social Desirability	0–1		–	–	0.60	0.27	.69
2. Affirming Beliefs	1–5	3.39	1.23	.92	3.17	1.27	.93
3. Cis/heteronormative Beliefs	1–5	1.85	0.92	.94	1.74	0.91	.94
4. CJCLDS Connectedness	1–4	1.95	0.97	.97	1.88	1.01	.97
5. Service Attendance	1–6	3.23	1.86	–	2.93	1.84	–
6. Family Support	1–5	3.40	1.03	.90	3.41	1.03	.92
7. Friend Support	1–5	3.85	0.99	.94	3.95	0.86	.91
8. Same-Gender Attraction	1–7	6.05	1.51	–	6.09	1.45	–
9. Other-Gender Attraction	1–7	2.01	1.43	–	2.00	0.54	–
10. Same-Gender Behavior	1–9	1.64	2.03	–	2.06	2.31	–
11. Other-Gender Behavior	1–9	0.89	1.71	–	0.91	1.83	–
12. Internalized Homonegativity	1–6	2.19	1.41	.90	2.18	1.34	.90
13. Outness	1–5	3.66	1.26	–	3.86	1.22	–
14. Self-Alienation	1–7	4.89	1.49	.87	5.07	1.39	.87
15. Depression	0–27	7.29	5.76	.88	6.12	5.22	.88
16. Suicidality	3–18	7.50	3.26	.73	6.75	3.49	.78
17. Flourishing	1–10	6.66	1.46	.91	–	–	–
18. Subjective Well-Being	1–7	–	–	–	5.57	0.97	.91
19. Self-Rated Health	1–5	3.47	0.94	–	3.41	0.97	–

We use the total scores for scales with meaningful clinical interpretative guidelines (i.e., Social Desirability, Depression, and Suicidality) but the average score for all other scales

We examined whether participants who engaged in SISOCE in 2022 or 2024 differed from those who did not in a variety of demographic variables to consider the inclusion of these variables as covariates in subsequent analyses. In 2022, participants still engaged in SISOCE did not differ from those not engaged in SISOCE in Gender χ^2^(4) = 4.77, *p* = .31, Race/Ethnicity χ^2^(1) = 0.62, *p* = .80, Education χ^2^(3) = 0.51 *p* = .92, *p* = .52, or Age *t*(317) = -.01, *p* = .99. In 2024, participants still engaged in SISOCE did not differ from those not engaged in SISOCE in Gender χ^2^(5) = 5.44, *p* = .37, Race/Ethnicity χ^2^(1) = 0.04, *p* = .85, Education χ^2^(3) = 2.16 *p* = .54, Age *t*(316) = −1.28, *p* = .10, or Desirable Responding *t*(310) = 1.26, *p* = .21. As such, no demographic variables were included as covariates in subsequent analyses.

### Differences in SISOCE Groups at Baseline

We next examined baseline differences between participants who reported ongoing or recently terminated SISOCE in 2022 and those who did not. We found that participants who reported ongoing or recently terminated SISOCE in 2022 were more likely to identify as Latter-day Saints (75.0%) than those who did not (46.2%; χ^2^(3) = 13.01, *p* < .05). We did not find differences in sexual identity between groups χ^2^(5) = 4.23, *p* = .52.

Omnibus MANOVA tests examining whether SISOCE groups differed in Religiousness, Support, Sexuality, and Health were universally significant (see Table 4). Post hoc tests revealed significant differences between groups in all variables tested except for in Affirming Beliefs and Suicidality. Participants who reported ongoing or recently terminated SISOCE reported higher religiousness, less support, more internalized minority stress, and worse health, most typically with small-to-medium effect sizes (Cohen, 1988). Taken together, our results suggest that participation or recent termination of SISOCE is clearly related to religiousness, support, internalized minority stress, and health. Clinically, the mean differences in Depression of the two groups is the difference between landing squarely in the categorization of “mild” depression (no SISOCE) and hitting the lower limit of “moderate” depression (SISOCE; Kroenke et al., 2001).

**Table 4:** Differences at baseline between participants who reported ongoing or recently terminated self-initiated sexual orientation change efforts and those who did not

		SISOCE *n* = 44	No SISOCE *n* = 272			
		*M* (*SD*)	*M* (*SD*)	*F*	η^2^	d
Religiousness			9.91^*^	.11		
	Service Attendance	4.30 (1.55)	3.05 (1.85)	17.89^*^	.05	−.70
	Affirming Beliefs	3.60 (1.09)	3.35 (1.25)	1.55	.01	−.21
	Cis/heteronormative Beliefs	2.56 (1.14)	1.74 (0.83)	32.43^*^	.09	−.93
	CJCLDS Connectedness	2.41 (0.89)	1.88 (0.96)	11.92^*^	.04	−.57
Support			3.24^*^	.02		
	Family Support	3.10 (0.90)	3.44 (1.05)	3.97^*^	.01	.32
	Friend Support	3.57 (0.98)	3.90 (0.98)	4.43^*^	.01	.34
Sexuality			12.25^*^	.18		
	Same-Gender Attraction	6.12 (1.15)	6.03 (1.58)	0.12	< .01	−.07
	Other-Gender Attraction	1.97 (1.45)	2.17 (1.31)	0.70	< .01	.14
	Outness	3.19 (1.27)	3.72 (1.27)	6.19^*^	.04	.42
	Internalized Homonegativity	3.69 (1.80)	2.02 (1.19)	58.26^*^	.17	−1.09
	Self-Alienation	4.27 (1.53)	5.07 (1.42)	10.86^*^	.02	.54
Health			3.17^*^	.04		
	Depression	9.72 (5.76)	6.93 (5.67)	3.51^*^	.03	−.48
	Suicidality	7.45 (3.28)	7.59 (3.11)	0.04	< .01	−.03
	Well-Being	6.09 (1.56)	6.74 (1.42)	16.31^*^	.02	.46
	Self-Rated Health	3.25 (0.97)	3.51 (0.93)	2.48^*^	.01	.27

^*^p < .05

### Differences in SISOCE Groups Two Years Later

We next examined whether engagement or recent termination of SISOCE in 2022 predicted participants’ religiousness, support, sexuality, or health two years later. Omnibus MANOVA tests examining whether SISOCE groups differed in Religiousness, Support, and Sexuality were universally significant (see Table 5). Post hoc tests revealed significant differences between groups on all variables tested except for Affirming Beliefs, Family Support, Same-Gender Attraction, and Other-Gender Attraction. Participants who reported ongoing or recently terminated SISOCE reported higher religiousness, less support, and more desire to be exclusively heterosexual (i.e., internalized homonegativity), most often with small-to-medium effect sizes (Cohen, 1988).

**Table 5:** Differences two years later between participants who reported ongoing or recently terminated self-initiated sexual orientation change efforts and those who did not

		SISOCE *n* = 44	No SISOCE *n* = 272			
		*M* (*SD*)	*M* (*SD*)	*F*	η^2^	d
Religiousness			5.34^*^	.07		
	Service Attendance	3.98 (1.75)	2.75 (1.80)	17.03^*^	.05	−.68
	Affirming Beliefs	3.50 (1.14)	3.13 (1.27)	3.21	.10	−.30
	Cis/heteronormative Beliefs	2.18 (1.19)	1.68 (0.85)	11.36^*^	.04	−.56
	CJCLDS Connectedness	2.39 (1.03)	1.79 (0.99)	13.26^*^	.04	−.60
Support			4.28^*^	.03		
	Family Support	3.17 (0.96)	3.44 (1.04)	2.57	.01	.26
	Friend Support	3.61 (0.86)	4.01 (0.86)	8.26^*^	.03	.47
Sexuality			6.44^*^	.11		
	Same-Gender Attraction	6.16 (1.17)	6.08 (1.51)	0.10	< .01	−.06
	Other-Gender Attraction	2.16 (1.44)	1.97 (1.48)	0.53	< .01	−.13
	Outness	3.29 (1.31)	3.88 (1.21)	7.53^*^	.03	.43
	Internalized Homonegativity	3.36 (1.73)	2.11 (1.23)	29.44^*^	.10	−1.12
	Self-Alienation	4.42 (1.52)	5.15 (1.37)	9.01^*^	.03	.37
Health			2.01	.03		
	Depression	7.83 (5.67)	5.85 (5.04)	5.89^*^	.02	−.39
	Suicidality	7.41 (2.92)	7.24 (3.37)	0.03	< .01	−.03
	Well-Being	5.37 (0.93)	5.60 (0.97)	2.13	.01	.24
	Self-Rated Health	3.39 (0.97)	3.40 (0.97)	0.01	< .01	.02

^*^p < .05

The omnibus MANOVA for Health was not significant, suggesting that we are not able to conclude that SISOCE impacts health broadly two years later. However, the ANOVA for Depression was significant and revealed a small-to-medium effect (*d* = .39), suggesting that participation in SISOCE in 2022 was related to increased Depression in 2024. Clinically, the mean differences between the two groups is the difference between landing squarely within the categorization of “mild” depression (no SISOCE) and reaching the upper limit of “mild” depression (SISOCE; Kroenke et al., 2001). Thus, we conclude that although we do not have evidence that SISOCE impacts health broadly two years later, there is evidence that engagement with SISOCE continues to impact Depression two years later.

### Changes in Sexuality Over Time

We next evaluated whether participants who were trying to change their sexual orientation reported changes in their sexual attraction, aversion, and/or behavior (see Table 6). The overall MANOVA was not significant, nor were the *F*-values for each of the six variables tested. Effect sizes ranged from unsubstantial to small (Cohen, 1988).

**Table 6:** Changes in sexual attraction, aversion, and behavior based on engagement with self-initiated sexual orientation change efforts

		SISOCE *n* = 39	No SISOCE *n* = 226			
		*M* (*SD*)	*M* (*SD*)	*F*	η^2^	d
Changes in Sexuality			0.86	.02		
	Changes in Same-Gender Attraction	0.04 (0.80)	−0.03 (0.67)	0.27	< .01	−.09
	Changes in Same-Gender Aversion	0.00 (1.05)	−0.10 (0.75)	0.34	< .01	−.11
	Changes in Same-Gender Behavior	0.42 (1.47)	0.46 (1.21)	0.02	< .01	.03
	Changes in Other-Gender Attraction	−0.02 (0.76)	0.18 (0.85)	2.16	.01	.25
	Changes in Other-Gender Aversion	0.11 (1.84)	0.13 (2.00)	0.01	< .01	.01
	Changes in Other-Gender Behavior	0.03 (0.96)	−0.15 (1.53)	0.96	< .01	−.14

^*^p < .05

Though not originally part of our analysis plan, we also conducted repeated measures ANOVA (see Table S1), allowing us to examine changes in sexuality within participants (i.e., do all participants report changes?), between participants (i.e., do participants in one group report differences from participants in the other group across time?), and interaction effects (i.e., do participants in one group change faster than participants in another group?). We also report Chi-squared analyses comparing the percentages of participants within each of the SISOCE groups reporting decreases, no change, and increases based on their engagement in SISOCE (see Table S2). These analyses supported our main analyses suggesting a lack of between-subjects, within-subjects, and interaction effects across all six indicators of sexuality, with one exception: within-subjects effects emerged for same-gender sexual behavior (*F* = 12.66, *p* < .01), with means suggesting that all participants engaged in more same-gender sexual behavior over time. Taken together, we cannot conclude that engaging in SISOCE led to changes in sexuality two years later.

## Discussion

With a sample of approximately 300 sexual minority current and former members of the CJCLDS who responded to surveys in 2022 and 2024, we examined who engaged in SISOCE, how engagement with SISOCE impacted participants’ well-being, and whether engagement in SISOCE led to changes in sexual orientation. Even though only a minority of participants reported recent SISOCE (n = 43; 13.7%), these participants differed markedly from those who did not report recent SISOCE both at baseline and two years later. The two groups did not differ in their experiences of change in their sexual orientation. We highlight four smaller takeaways that coalesce into a fifth, broader takeaway. As we do so, we note that our sample was unique in many ways (e.g., all come from an LDS background, the majority identify as LGBTQ+ rather than rejecting an LGBTQ+ label, small *n*) and as such encourage interpretation and generalization of our findings to be done within those limitations in mind.

### Engaging in SISOCE Is Common But Sustaining It Is Rare

We found that although most of our religiously raised participants engaged in SISOCE (74.6%) at some point in their lives, relatively few were engaged in SISOCE at the start of the study (13.7%), and even fewer were engaged in SISOCE after two years (5.1%). Together, these frequencies suggest that engaging in SISOCE is common among our participants but that sustaining it is rare.

Although our data suggest that sustaining SISOCE is rare, it is less clear exactly why so many participants stopped engaging with SISOCE. One explanation is that participants may have either experienced a lack of change from SISOCE or experienced harm from SISOCE, as many LGBTQ+ people have reported in previous work (Blosnich et al., 2020; Tran et al., 2024). Methodologically, however, we did not examine whether SISOCE itself was the cause for participants’ cessation of SISOCE, and as such, it is possible that participants may have stopped engaging with SISOCE because of other life changes such as aging, broader sexual identity development, or loss of SISOCE-specific support networks.

### SISOCE Do Not Appear to Change Sexual Orientation

One reason why participants may stop their SISOCE is because they experience meaningful shifts in their sexual orientation; however, our data suggest that these shifts are unlikely. When comparing participants who had engaged in SISOCE to those who had not, we failed to find significant or substantial differences between groups in sexual attraction, sexual aversion, or sexual behavior. These findings support others who have found SISOCE to be ineffective in changing sexual orientation (Comer et al., 2024; Przeworski et al., 2021).

The prospective and longitudinal nature of this study makes the null findings more likely to be of import. Studies that have found change from SISOCE have largely relied on cross-sectional surveys that explicitly ask participants if they have experienced change in their orientation (Byrd et al., 2008; Nicolosi et al., 2000). Because people who engage in SISOCE by definition are hoping that their sexual orientation will change, they may be more likely to report change in order to confirm their hopes, leading researchers to find an “effect” for SISOCE where this “effect” may reflect participants hope to change or desire to change. In contrast, we asked our participants to report the degree to which they experience attraction and aversion to men and women at baseline and two years later, eliminating much of the possibility for a desirability bias. Thus, the lack of change in concrete, anchored measures of sexual attraction and aversion is likely a reflection of a true lack of change in sexual orientation.

### SISOCE Is Often Religiously Motivated

The largest effects that we observed between our religiously raised participants who were and were not engaged in SISOCE were in participants’ religiousness and desires to be exclusively heterosexual (i.e., internalized homonegativity). Participants engaged in SISOCE reported attending religious services more often, holding more cis/heteronormative religious beliefs, and being more connected with conservative religious communities than those not engaged in SISOCE, at both baseline and longitudinally. These participants also greatly differed in how much they accepted and how positively they viewed their same-gender sexual attraction.

Many conservative religious teachings promote a worldview where same-gender sexuality is a challenge or trial at best and a premise for damnation at worst. This pattern is particularly true in the context of the CJCLDS, where ideals about heterosexual marriage and childbearing are enshrined in views of salvation and eternity (Lefevor et al., 2022a, 2022b; McGraw et al., 2023). For conservatively religious participants, engaging in SISOCE may be a way to maintain religious congruence and not risk temporal (e.g., rejection) or eternal (e.g., damnation) consequences associated with same-gender sexuality. Thus, even if SISOCE are not formally promoted by the conservative religious organization, the heteronormative values likely create an environment where SISOCE emerge.

There are robust meta-analytic findings linking concealment (Pachankis et al., 2020) and internalized homonegativity (Lefevor et al., 2023a, 2023b) to negative health outcomes. Conversely, meta-analytic results suggest that religiousness is largely positively related to health for sexual minorities (Lefevor et al., 2021). When the differences between our two SISOCE groups in outness, internalized homonegativity, and religiousness are understood in light of these health differences, the plight of LGBTQ+ Latter-day Saints becomes even clearer: remaining religiously affiliated may provide access to religious coping resources but likely comes at the cost of internalized stigma and decreased outness. Conversely, religiously disaffiliating comes at the cost of increase stress from a faith transition but may ultimately lead to more outness and less internalized stigma (Lefevor et al., 2023a, 2023b).

### Engaging in SISOCE Has Adverse Health Impacts

At baseline, we found that participants engaged in SISOCE reported more depression, less well-being, less self-rated health, and less family and friend support (though similar degrees of suicidality) than those not engaged in SISOCE. More notably, we found that some of these differences persisted longitudinally, with participants engaged with SISOCE at baseline reporting less friend support and more depression than those not engaged with SISOCE two years later. These disparities point attention to the fact that *trying* to change one’s orientation can negatively impact health and also provide some insight into why this is.

Firstly, we note that many of the adverse health impacts of engagement with SISOCE appeared to be present only while participants were trying to change. Differences in well-being, self-rated health, and family support existed at baseline but not longitudinally, suggesting that the act of *trying to change* may undermine health; as such, cross-sectional group differences may be understood as differences between participants who were trying to change their sexual orientation and those who did not. Further, these two groups differed in their degree of acceptance of same-gender attractions—with those engaged in SISOCE being much less accepting of their attractions than those not engaged—suggesting that failing to accept one’s experience of same-gender sexuality may be at least partially responsible for group differences in well-being, self-rated health, and support.

Secondly, we note that group differences in depression and friend support persisted two years later, even when the majority of the group who was pursuing SISOCE at baseline had stopped pursuing SISOCE. These findings suggest that while much of the distress of SISOCE for our participants may be related to currently trying to change, trying to change may have downstream consequences. The exact mechanisms of this distress are not clear, but it may be that trying to change may delay developmental processes (e.g., questioning fears of rejection and negative beliefs about oneself and same-gender sexuality, building pair bonding-related social skills), which in turn make it harder to connect with others and may create an environment where depressive symptoms persist. Similarly and conversely, delayed developmental processes (e.g., persistent fears of rejection and negative beliefs about oneself and same-gender sexuality, poor social skills), which may be fueled by difficulty acknowledging one’s internal experience, may also lead individuals to pursue SISOCE (Drescher & Byne, 2024). We particularly note that our measure of depression examined depressive symptoms over the past two weeks while our measure of SISOCE was a lifetime measure. Particularly without any “pre-SISOCE” measures of depression, this disparity makes it particularly difficult to draw causal conclusions about the relationship between SISOCE and depression.

At the same time, we note that there are several other potential explanations for these changes. The period between the two time points was during the COVID-19 pandemic, and it is possible that social isolation more clearly impacted participants engaging in SISOCE than those not engaging in SISOCE. Similarly, both groups of participants may have had difficulty accessing mental health treatment in this timeframe, so the magnitude of group differences may be smaller.

### Engaging in SISOCE May Be Developmental in Heteronormative Social Milieus

We found that participants engaged in SISOCE were less out, had more desires to be exclusively heterosexual (i.e., internalized homonegativity), and were more self-alienated than those not engaged in SISOCE. When paired with findings about how commonly participants pursued SISOCE and then stopped pursuing SISOCE, these findings suggest that engaging in SISOCE may be developmental, at least for sexual minorities raised in a conservative religion (Drescher & Byne, 2024). It may thus be typical for sexual minority individuals raised in a conservative religion to initially react to their same-gender sexual attractions by trying to change them in an effort to follow conservative religious and social norms about marriage and family or to avoid the discrimination faced by many LGBQ+ individuals (Meyer et al., 2021). For these individuals, only when change efforts fail would they start to make the cognitive and behavioral shifts necessary to live a flourishing life as an LGBQ+ individual, questioning fears of rejection and negative beliefs about oneself and same-gender sexuality. Indeed, early models of sexual identity development often described initial stages characterized by confusion and change seeking (Cass, 1979), noting that after self-awareness of one’s same-gender sexuality, people tend to either identify as LGBTQ+ or reject an LGBQ+ label (Drescher & Byne, 2024).

The fact that engaging in SISOCE may be developmental does not mean that engaging in SISOCE is a *necessary* or even helpful step; rather, we suggest that it is a *typical* step in sexual identity development. Likely, societal and religious norms about marriage and family that suggest that exclusive heterosexuality is the ideal (i.e., heteronormativity) are responsible for sexual minority participants’ engagement with SISOCE. Disengaging from SISOCE may reflect a vision for flourishing outside of those societal and religious norms.

### Limitations

Our findings have several limitations. Firstly, our participants had a shared and quite specific religious, gender, and racialized identity (most were White, cisgender, adult men raised in the CJCLDS). It is not clear which results are attributable to our unique sample; however, we imagine that some associations would function differently in a different sample (e.g., adolescents may be more affected by SISOCE than adults). Secondly, our sample was relatively small, with only 43 participants reporting ongoing or recently terminated SISOCE. This limitation also made it more difficult to examine gender differences in our sample. Thirdly, the differences we observed between groups—though often significant—had modest clinical significance. In particular, longitudinal group differences in Depression were about two points on the PHQ-9, effectively shifting participants’ average scores from the middle of the “mild” depression category to the top of the “mild depression” category. Fourth, our sample largely identified as LGBQ+ at the outset of the study, which may reflect that many participants had already rejected change efforts. As such, the prevalence of and length of engagement with SISOCE in our sample may not accurately represent that of all sexual minorities. Fifth, our sample was largely monosexual, and greater sexual fluidity has been reported in more bisexually oriented individuals (Sullins & Rosik, 2025). Similarly, we had some participants who identified as transgender/nonbinary, and it is not clear how/why participants determined that the question about SISOCE was relevant to them since it was framed in language about binary sexual orientation. Sixth, we had a relatively poor retention rate in our study (57%). Although we examined differences between participants who did and did not return to our study, it is concerning that so few returned. Seventh, we ultimately relied on self-report data, which means that participants’ reports of both their engagement with SISOCE and mental health may be susceptible to biased recall, inaccurate reporting, and heterogeneity of experiences of SISOCE (e.g., some participants likely only experienced personal efforts to change their orientation where others went to organized, group change efforts). However, we note that groups did not differ in socially desirable responding. Eighth, we compared SISOCE undergone by individuals that were heterogeneous in nature (e.g., some participants may have engaged in SISOCE through avoidance where others may have gone to self-help groups) and we know relatively little about the frequency or intensity of these efforts. All of these limitations severely impede our ability to make causal conclusions from our data.

### Conclusions

We created an ideologically diverse research team, preregistered our hypotheses, and used a prospective, longitudinal design to produce data that could inform future scientific and policy endeavors. In our sample of 315 sexual minority individuals, we found that the majority engaged in SISOCE at some point in their lives but ultimately desisted or met their goals. We failed to find evidence that engaging in SISOCE led to a significant change in sexual orientation. We found that participants who engaged in SISOCE reported less support and more depression than participants who did not engage in SISOCE, though we did not find significant differences between groups in suicidality. We suggest that—due to societal heteronormativity—engagement in SISOCE is often a step in sexual minorities’ sexual identity development, a step that can be characterized by struggle and pain.

## Supplementary Information

Below is the link to the electronic supplementary material.

Supplementary file (DOCX 15 KB)

## Data Availability

See https://osf.io/ta2pw/?view_only=19437655f78f41569020d12ba1529b33 for the preregistered method section, which includes research questions, procedures, participants, measures, data, code, and analysis plan.
