# Supplementary material for: A Preregistered, Prospective, Longitudinal Examination of the Impact of Self-Initiated Sexual Orientation Change Efforts on Health and Changes in Sexuality
Source: Arch Sex Behav. 2026 Aug 15;55(6):2773–89. doi: 10.1007/s10508-026-03517-y (PMC13597570; doi:10.1007/s10508-026-03517-y)
Supplement: Supplementary file 1 [file 10508_2026_3517_MOESM1_ESM.docx]

Online Supplemental Materials

**Table S1**

*Changes in Sexual Attraction, Aversion, and Behavior Based on Engagement with Self-Initiated Sexual Orientation Change Efforts*

| Variable | SISOCE (*n* = 39) | | No SISOCE (*n* = 226) | |  |  |  |
| --- | --- | --- | --- | --- | --- | --- | --- |
|  | W1 *M* (*SD*) | W1 *M* (*SD*) | W1 *M* (*SD*) | W1 *M* (*SD*) | *F_bet_* | *F*_with_ | *F*_int_ |
| Same-Gender Sexual Attraction | 6.21 (0.24) | 6.18 (0.23) | 6.06 (0.10) | 6.10 (0.10) | 0.21 | 0.02 | 0.27 |
| Same-Gender Sexual Aversion | 1.56 (0.16) | 1.46 (0.17) | 1.46 (0.07) | 1.46 (0.07) | 0.11 | 0.34 | 0.34 |
| Same-Gender Sexual Behavior | 1.39 (0.33) | 1.85 (0.37) | 1.72 (0.14) | 2.15 (0.16) | 0.78 | 12.66^*^ | 0.22 |
| Other-Gender Sexual Attraction | 1.97 (0.22) | 2.15 (0.23) | 1.95 (0.09) | 1.93 (0.10) | 0.29 | 1.45 | 2.16 |
| Other-Gender Sexual Aversion | 3.85 (0.34) | 3.97 (0.34) | 3.84 (0.14) | 3.95 (0.14) | 0.53 | 0.01 | 0.01 |
| Other-Gender Sexual Behavior | 1.00 (0.27) | 0.85 (0.28) | 0.83 (0.11) | 0.85 (0.12) | 0.48 | 0.96 | 0.09 |

*Note*: ^*^*p* < .05; *F*_bet_ = *F*_between_; *F*_with_ = *F*_within_; *F*_int_ = *F*_interaction_; W1 = Wave I; W2 = Wave II

**Table S2**

*Percentages of Participants Reporting Changes in Sexual Attraction, Aversion, and Behavior Based on Engagement with Self-Initiated Sexual Orientation Change Efforts*

|  |  | SISOCE  *n* = 39 | | | No SISOCE  *n* = 226 | | |  |  |
| --- | --- | --- | --- | --- | --- | --- | --- | --- | --- |
|  |  | *% Less* | *% Same* | *% More* | *% Less* | *% Same* | *% More* | *χ^2^* | V |
| Changes in Sexuality | |  |  |  |  |  |  |  |  |
|  | Changes in Same-Gender Attraction | 0.0% | 97.4% | 2.6% | 2.2% | 93.8% | 4.0% | 1.09 | .06 |
|  | Changes in Same-Gender Aversion | 7.7% | 92.3% | 0.0% | 4.4% | 92.0% | 3.5% | 2.11 | .09 |
|  | Changes in Same-Gender Behavior | 5.1% | 71.8% | 23.1% | 4.9% | 78.3% | 16.8% | 0.92 | .06 |
|  | Changes in Other-Gender Attraction | 2.6% | 87.2% | 10.3% | 3.5% | 93.4% | 3.1% | 4.34 | .13 |
|  | Changes in Other-Gender Aversion | 15.4% | 69.2% | 15.4% | 14.6% | 69.9% | 15.5% | 0.02 | .01 |
|  | Changes in Other-Gender Behavior | 10.3% | 87.2% | 2.6% | 4.0% | 92.9% | 3.1% | 2.82 | .10 |

*Note*: ^*^*p* < .05; A change of greater than one scale point was classified as “less” or “more” depending on direction
